# Nutrition Therapy for Liver Diseases Based on the Status of Nutritional Intake

**DOI:** 10.1155/2012/859697

**Published:** 2012-11-14

**Authors:** Kenichiro Yasutake, Motoyuki Kohjima, Manabu Nakashima, Kazuhiro Kotoh, Makoto Nakamuta, Munechika Enjoji

**Affiliations:** ^1^Department of Health and Nutrition Sciences, Faculty of Health and Social Welfare Sciences, Nishikyushu University, Kanzaki 842-8585, Japan; ^2^Clinical Research Center, Kyushu Medical Center, National Hospital Organization, Fukuoka 810-0065, Japan; ^3^Department of Gastroenterology, Kyushu Medical Center, National Hospital Organization, Fukuoka 810-0065, Japan; ^4^Health Care Center and Faculty of Pharmaceutical Sciences, Fukuoka University, Fukuoka 814-0180, Japan; ^5^Department of Medicine and Bioregulatory Science, Graduate School of Medical Sciences, Kyushu University, Fukuoka 812-8582, Japan

## Abstract

The dietary intake of patients with nonalcoholic fatty liver disease (NAFLD) is generally characterized by high levels of carbohydrate, fat, and/or cholesterol, and these dietary patterns influence hepatic lipid metabolism in the patients. Therefore, careful investigation of dietary habits could lead to better nutrition therapy in NAFLD patients. The main treatment for chronic hepatitis C (CHC) is interferon-based antiviral therapy, which often causes a decrease in appetite and energy intake; hence, nutritional support is also required during therapy to prevent undernourishment, treatment interruption, and a reduction in quality of life. Moreover, addition of some nutrients that act to suppress viral proliferation is recommended. As a substitutive treatment, low-iron diet therapy, which is relatively safe and effective for preventing hepatocellular carcinoma, is also recommended for CHC patients. Some patients with liver cirrhosis (LC) have decreased dietary energy and protein intake, while the number of LC patients with overeating and obesity is increasing, indicating that the nutritional state of LC patients has a broad spectrum. Therefore, nutrition therapy for LC patients should be planned on an assessment of their complications, nutritional state, and dietary intake. Late evening snacks, branched-chain amino acids, zinc, and probiotics are considered for effective nutritional utilization.

## 1. Introduction

The liver is one of the main organs of nutritional metabolism, including protein synthesis, glycogen storage, and detoxification. These functions become damaged to a greater or lesser extent in patients with liver diseases, resulting in various metabolic disorders, and their disturbed nutritional condition is associated with disease progression. Therefore, dietary counseling and nutritional intervention can support other medical treatments in some liver diseases.

Nonalcoholic fatty liver disease (NAFLD) is a disease caused by excessive dietary intake, which leads to hepatocytic triglyceride accumulation, obesity, and insulin resistance; hence, nutrition therapy is a basic treatment for NAFLD. NAFLD has a wide spectrum of pathologic conditions from simple steatosis to steatosis with necroinflammation and fibrosis, the condition termed nonalcoholic steatohepatitis (NASH). Nutritional intake in NAFLD patients is characterized as energy overload by a high-carbohydrate and high-fat diet, or excessive cholesterol intake. In patients with chronic hepatitis C (CHC), nutritional support is expected to promote the effect of antiviral treatment, for example, n-3 polyunsaturated fatty acids (PUFAs) inhibit HCV replication, and a low-iron diet is effective in reducing hepatic injury. Various nutritional problems as well as clinical symptoms lie in liver cirrhosis (LC), the end stage of chronic hepatitis, complications of influence and prognosis. Therefore, nutrition therapy is important in preventing these problems. In this paper, nutritional aspects and beneficial nutrition therapies are outlined in patients with NAFLD/NASH, CHC, and LC.

## 2. Profile of Nutritional Intake in NAFLD Patients

### 2.1. High-Carbohydrate Diet Including Excessive Intake of Soft Drinks

Studies of NAFLD patients found that they had an increased daily consumption of sugar or sugar-containing beverages by twice or more when compared with their matched controls [1–3]. Imaging indicated that fatty liver disease worsened with an increase in the number of bottles of soft drinks consumed, suggesting that consumption of sugar-containing beverages is a significant predictor of NAFLD [3]. Moreover, in NASH patients, the percentage of simple sugars or carbohydrates contributing to total energy intake was considerably higher compared with that in simple steatosis patients [4]. These findings are explained by the following mechanism; excessive carbohydrates/sugar intake activates sterol regulatory element-binding protein-1c (SREBP-1c), which acts as a transcription factor to activate *de novo* fatty acid synthesis in hepatocytes [5]. 

### 2.2. High-Fat Diet 

 It has been recognized that energy overload by excessive fat intake causes NAFLD [6]. When dietary habits were compared between NASH patients and healthy individuals, the intake of saturated fatty acids was found to be significantly higher in NASH patients [7]. In model animals with an equivalent daily calorie intake, increasing the fat/energy ratio with a high-fat diet resulted in an increase in body weight, upregulation of blood glucose levels, progression of steatosis, and marked inflammation of the liver [8], indicating a close association between excessive fat intake and NASH. In this regard, it is proposed that peroxisome proliferator-activated receptor-*γ*  (PPAR-*γ*) activation may play an important role [5]. 

### 2.3. Excessive Cholesterol Intake 

In an investigation of nutritional intake, dietary cholesterol levels were significantly higher in NASH patients compared with healthy individuals [7]. Also in our study, dietary cholesterol levels were significantly higher in the order of nonobese NAFLD patients, obese NAFLD patients, and healthy controls [9]. In animal models, in which dietary energy intake was within normal limits, a high-cholesterol diet induced NAFLD without obesity [10–12]. Excessive cholesterol intake leads to an increase in its metabolites, oxysterols, which are agonistic ligands for liver X receptor  *α*  (LXR*α*), resulting in activation of the LXR*α*-SREBP-1c pathway and *de novo* fatty acid synthesis in hepatocytes [13, 14]. Furthermore, in the NAFLD liver, hepatocytic cholesterol is excessive but *de novo* cholesterol synthesis is further activated hence, lipid metabolism is dysregulated [13, 14]. 

## 3. Nutrition Therapy for NAFLD Patients

### 3.1. Treatment for Obese Patients (Ordinary Type of NAFLD) 

 When nutrition therapy is considered for NAFLD patients, the actual nutritional intake and content should first be examined in detail to determine which nutrient is the main cause of NAFLD, that is, carbohydrates, fat, or cholesterol. Because the state of nutritional intake and hepatic expression patterns of lipid metabolism-associated factors are different between obese and nonobese NAFLD patients, the target of nutrition therapy is also different in the two groups. 

It is common knowledge that the main cause of NAFLD is excessive dietary energy intake in obese patients. In practice, a reduction in nutritional intake by metabolic surgery or dietary counseling improves the condition of NAFLD in obese patients [15, 16]. Usually, the profile of nutritional intake in obese NAFLD patients shows excessive intake of carbohydrates and fat. Therefore, normalizing their intake of these nutrients, which leads to weight reduction, can correct a vicious cycle of abnormal hepatic lipid metabolism. However, it is often hard for patients to maintain weight reduction. Additional therapies for weight reduction, including inhibitors against gastric and pancreatic lipases, such as orlistat, and new antagonists against endocannabinoid receptors, are now being developed although these have not proved to be effective or without side effects. 

As additional nutritional means, PUFAs and vitamin E with antioxidant effects may be effective in NAFLD. In a clinical trial of NAFLD patients, treatment with ethyl icosapentate, a type of n-3 PUFA, for 12 months improved liver function to some extent in a biochemical evaluation [17]. n-3 PUFAs exhibit their effect by suppressing the activity of SREBP-1c and *de novo* fatty acid synthesis. It means that n-3 PUFAs may be effective for patients in whom the main cause of NAFLD is excessive intake of carbohydrates or a shortage of PUFAs, but less effective in NAFLD caused by excessive fat intake. Vitamin E exhibits a greater effect in patients with NASH compared with patients with simple steatosis because of its strong antioxidant activity [18]. 

### 3.2. Treatment for Nonobese Patients 

A substantial proportion of NAFLD patients are nonobese and/or are without insulin-resistance in Japan [19, 20]. In our study of nutritional intake, mean intake levels of proteins, fat, carbohydrates, and total energy were not excessive in nonobese patients [9]. However, dietary cholesterol intake was markedly excessive, while intake of PUFAs was insufficient in nonobese patients compared with obese patients and healthy individuals [9]. These characteristic findings may be closely associated with the pathogenesis of NAFLD. In our study, expression levels of lipogenic transcription factor LXR*α*, of which agonistic ligands are oxysterols, were significantly higher in nonobese patients compared with obese patients [13, 14]. Thus, in nonobese NAFLD patients, an excess of cholesterol and its metabolites (oxysterols), leading to activation of *de novo* fatty acid synthesis via the LXR*α*-SREBP-1c pathway, should be considered as a main cause of steatosis. As a nutritional treatment for these patients, intake of food containing a high level of cholesterol should be restricted. Accordingly, a Niemann-Pick C1-like 1 (NPC1L1) inhibitor (ezetimibe), which decreases cholesterol absorption in the intestine, is a reasonable treatment relevant to nutrition therapy. In practice, ezetimibe treatment in nonobese NAFLD patients improves liver injury and steatosis [21]. Also in animal models, inactivation of NPC1L1, a critical mediator of cholesterol absorption, shows protective effects against diet-induced hypercholesterolemia and fatty liver, and ezetimibe treatment improves liver steatosis and insulin resistance in obese rat models [22, 23]. 

n-3 PUFAs can improve insulin resistance and NAFLD by lowering the hepatic tumor necrosis factor  *α*  level. n-3 PUFAs also suppress fatty acid synthesis by controlling SREBP-1c expression negatively and promote fatty acid  *β*-oxidation by activating PPAR*α*  expression [24, 25]. These facts suggest the possibility that a shortage of PUFA intake leads to NAFLD independent of dietary energy intake. Because a shortage of PUFA intake is found in nonobese NAFLD patients, n-3 PUFA-rich fish and supplements of n-3 PUFAs, such as EPA and docosahexaenoic acid, are recommended as nutrition therapy. Some herbal compounds, such as curcuma, have antioxidant effects and they are expected to show therapeutic effects on NAFLD. 

## 4. Profile of Nutritional Intake in CHC Patients 

Presently, the main strategy for CHC is interferon (IFN)-based antiviral therapy and liver protective treatments. In Japan, a high energy, high protein, and high vitamin diet was previously recommended for CHC patients. This principle had spread and become established by broadening the meaning of nutrition therapy reported by Patek and Post, which was adequate for improving the prognosis of alcoholic hepatitis in heavy drinkers [26]. Generally, the nutritional intake of CHC patients is almost similar to that of healthy individuals [27]. However, during IFN-based antiviral treatment, weight loss is apparent in 11–29% of patients because of decreased appetite and malnutrition [28–32]. Although reducing iron by phlebotomy and a low iron diet are also effective for liver injury in CHC patients [33–35], some patients still take iron-rich food and supplements because they believe the incorrect information that glycogen- and iron-rich corbiculae, curcuma, and bovine liver, which are traditional food used to treat acute hepatitis in folk remedies, show therapeutic effects for chronic hepatitis. 

## 5. Nutrition Therapy for CHC Patients

### 5.1. Nutrition Therapy during IFN-Based Antiviral Therapy 

 In CHC patients, HCV infection causes a disturbance of glucose and lipid metabolism, and liver steatosis [36–38], which reduces the effect by IFN-based antiviral therapy and affects liver fibrosis [39–42]. However, some patients have a glucose and lipid metabolism disorder, and obesity due to lifestyle-related disease independent of HCV infection. 

A recent meta-analysis indicated that insulin resistance reduces the antiviral effect of IFN-based therapy, regardless of HCV genotype [43, 44]. Therefore, before starting antiviral therapy, it is better to improve some metabolic disorders including obesity and insulin resistance by diet therapy and exercise therapy. After starting IFN-based therapy, body weight decreases due to decreased appetite and some digestive symptoms in many patients [28, 29]. A recent study showed that resting energy expenditure did not increase during IFN-based antiviral therapy, and weight loss during the therapy was ascribed to a decrease in energy intake [29]. Although there are no guidelines on how nutrition therapy should be conducted, a decrease in quality of life or malnutrition must be prevented for avoiding an interruption in antiviral therapy.

Various nutrients have lately been identified to be associated with suppression or promotion of HCV proliferation and attract considerable notice [45]. It is known that  *β*-carotene, vitamin D, linoleic acid, arachidonic acid, eicosapentaenoic acid, docosahexaenoic acid, iron, and zinc have suppressive effects, while retinol, vitamin E, vitamin K, vitamin C, cholesterol, and selenium have promoting effects on HCV proliferation. For example, when the serum concentration of vitamin D was maintained at 32 ng/mL or more by daily administration of 2,000 IU vitamin D_3_, the antiviral effect of IFN-based treatment in CHC patients was markedly improved [46]. There is a fair possibility that a shortage of PUFA intake worsens the antiviral effect of IFN-based treatment in CHC patients [47]. Also, in an *in vitro* study, HCV proliferation was markedly suppressed by treatment with PUFAs, such as arachidonic acid, eicosapentaenoic acid, and docosahexaenoic acid [48, 49]. Additionally, it has been reported that  *β*-carotene-containing food and herbal food show an adjuvant effect for IFN-based antiviral therapy [50, 51]. Accordingly, these nutrients are expected to be used as adjuvants in antiviral therapy for patients with HCV infection. 

### 5.2. Nutrition Therapy for Patients with Iron Accumulation 

 In CHC patients, hepatic iron uptake is accelerated and excessive iron accumulation is often apparent in hepatocytes [52–59]. When excessive iron changes to Fe^3+^ from Fe^2+^, free radicals are produced and the oxidative stress causes injury of cell-membrane and DNA, leading to hepatitis progression. To prevent the iron-mediated injury, phlebotomy and low iron diet therapy is effective [33–35]. Long-term phlebotomy with low-iron diet therapy lowers the risk of development of hepatocellular carcinoma (HCC) from CHC [60]. In Japan, the desired daily iron intake is <6 g in low-iron diet therapy. There are two forms of dietary iron: heme iron, which is present in fish and meat, and nonheme iron, which is present in vegetables. The absorption rate of iron is 10–40% in fish and meat, and 0.3–5% in vegetables; therefore, dietary intake of heme iron must be especially considered in dietary counseling. It is known that a considerable quantity of iron is contained in lean meat, red flesh, internal organs, and the dark flesh of a fish; thus, the specific foods to eat should be specified. However, in low-iron diet therapy, too great a limitation of dietary intake or food selection can result in total nutritional imbalance. Therefore, regular dietary monitoring is required for low-iron diet therapy. 

## 6. Profile of Nutritional Intake in LC Patients

LC is a consequence of all forms of chronic hepatic injury characterized by destruction of hepatic architecture and vascular structures with deposition of fibrotic tissue, which leads to functional decompensation. In some LC patients, intake of various nutrients decreases, which accelerates hepatic dysfunction including a metabolic disorder, resulting in protein energy malnutrition (PEM) [61–65]. Some investigations have shown that LC patients have a trend to take more energy via carbohydrates, which may reflect their insufficient glycogen storage, and fasting accelerates the oxidation of fat [66–68]. 

A recent increase in the obese population is a difficult worldwide problem, and the increase is also marked in LC patients [69]. This phenomenon indicates that a previous trend of malnutrition has changed into excessive energy intake in LC patients. Recently, there has been an increase in NASH causing LC. As described above, nutrition therapy for NASH patients is to correct their excessive dietary energy intake. Also, at present, in LC caused by alcohol and HCV infection, nutritional intake leans toward being either sufficient or excessive [68, 70]. In our study of compensated LC patients with HCV infection, dietary energy intake and/or protein intake were excessive in 70% of patients and their intake of energy, proteins, and fat was significantly higher than that of healthy individuals [70]. Mean body mass index (BMI) of patients with NASH causing LC was 27.6 kg/m^2^, while more than 30% of patients with viral hepatitis causing LC were also obese (BMI > 25 kg/m^2^) [69]. Although the nutritional state of LC patients cannot be estimated by BMI alone, a recent trend of increased nutritional intake is apparent in LC patients. The nutritional state of LC patients has become diversified by recent changes in dietary habits and by the progression of nutrition therapy, including treatment with branched-chain amino acids (BCAAs). 

## 7. Nutrition Therapy for LC Patients

### 7.1. Nutrition Therapy for LC with PEM 

As a guideline for energy intake and protein intake, the European Society for Clinical Nutrition and Metabolism (ESPEN) advocated the consensus nutrition standard for LC patients: 35–40 kcal/kg/day in energy and 1.2–1.5 kcal/kg/day in proteins [71]. However, the standard is not always pertinent and should be altered depending on conditions, such as race, intensity of daily activity, PEM, glucose intolerance, protein intolerance, and obesity. Therefore, flexible handling of the guideline is needed. For nutritional assessment of patients with LC, calorimetry may be the best way.

Regarding pathophysiology of PEM in LC patients, appetite stimulation signals from the hypothalamus are suppressed via downregulation of cholecystokinin clearance or cytokine secretion from internal organs, and additionally, ascitic fluid/intestinal edema induces appetite loss [61–63]. Moreover, in a metabolic disorder in LC, resting energy expenditure is upregulated and accompanied by an increased combustion rate of fat, resulting in downregulation of the nonprotein respiratory quotient. These changes are explained by a reduction in hepatic functional reserve, glycogen storage, and insulin sensitivity. In protein metabolism, serum BCAA values decrease markedly because of increased BCAA consumption in skeletal muscle as a substrate for efficient energy production and a substrate for compensatory metabolization of ammonia [72, 73]. When energy nutrition and protein nutrition in viral LC patients were assessed by indirect calorimetry and serum albumin level, respectively, 62% of patients had energy malnutrition, 75% of patients had protein malnutrition, and 50% of patients had energy and protein malnutrition [65]. As a measure for energy malnutrition, a late evening snack (LES) is recommended. When the number of meals is divided into 4–6 per day, nitrogen balance improves [74]. Also glucide intake at night shows a similar effect [75]. The disruption of muscle protein is suppressed by BCAA intake at night and glucose tolerability is improved by BCAA with glucide intake at night [76, 77]. Because a simple LES addition induces energy overload promoting obesity and glucose intolerance, it is important that <200 kcal are allocated to the LES from the standard daily total energy intake. Concrete examples of LES are a snack mainly consisting of carbohydrates, general enteral nutrients, BCAA-rich enteral nutrition, and so on [76, 78–82]. It has been reported in LC patients with LES that energy metabolism (respiration quotient), serum-free fatty acid levels, and urine 3-methylhystidine levels improve in a week, and serum albumin levels, nitrogen balance, and QOL improve within 3 months [76, 78, 79]. 

The incidence of protein metabolism disorder is high in LC, and the disorder becomes more marked with the progression of cirrhosis. BCAA-rich enteral nutrition or oral BCAA granules are used for protein metabolism disorder in order to improve nitrogen balance and undernutrition. BCAA-rich enteral nutrition is adequate for patients with chronic hepatic failure, protein intolerance, and a history of hepatic encephalopathy, and BCAA granules are suitable for patients with adequate dietary intake but with hypoalbuminemia resulting from the protein metabolism disorder. In studies in decompensated LC patients, there was a high evidence level that BCAA-rich enteral nutrition and BCAA granules improved hypoalbuminemia, edema, ascitic fluid, event-free survival rates, and QOL [69, 78, 83–85]. However, the clinical response to BCAA was better at an early stage of hepatic failure [86]. 

PEM is based on the metabolic disorder and, at present, LES and oral BCAA supplementation are strongly recommended after assessment of the metabolic disorder and severity of malnutrition. However, more evidence is being accumulated on an ordinary diet therapy for LC patients with PEM, and new standard may be presented hereafter. 

### 7.2. Nutrition Therapy for LC with Glucose Intolerance 

 Insulin resistance/hyperinsulinemia and glucose intolerance are often shown in LC patients and are associated with a reduction in glucose uptake in the liver and peripheral tissues [87]. It is nutritionally important that improving hyperinsulinemia brings about normalization of insulin-dependent glucose uptake and glycogen synthesis [88]. Nutrition therapy for LC patients with glucose intolerance requires a lower standard of energy intake to prevent hyperinsulinemia and hyperglycemia. In Japan, the standard of 25–30 kcal/kg ideal body weight/day is an advisable range. Dietary fiber-rich meals with a low glycemic index, a lower content of simple carbohydrates, and more exercise, as well as  *α*-glucosidase inhibitor (*α*-GI) or insulin with  *α*-GI treatment, improve hyperinsulinemia and hyperglycemia in LC patients [89–92]. Zinc supplementation is also effective for improving hyperglycemia [93]. 

### 7.3. Nutrition Therapy for LC with a History of Hepatic Encephalopathy 

Hepatic encephalopathy is caused by highly impaired hepatic function and portosystemic shunt formation. Hepatic disruption of ammonia processing and urea synthesis is ascribed to hyperammonemia and a decrease in Fischer's ratio and BCAA/Tyr ratio. Nutritional induction factors of hepatic encephalopathy are excessive intake of dietary proteins and constipation. A protein-restricted diet of <40 g/day and BCAA supplementation has been generally recommended for patients with episodic hepatic encephalopathy or decompensated cirrhosis [94]. Because long-term protein restriction promotes catabolism of body proteins and PEM, it must be combined with BCAA supplementation. Increasing the intake of insoluble dietary fiber-rich vegetables serves to improve and prevent constipation. However, it is difficult to control serum ammonia levels by diet therapies alone, and synthetic disaccharides, such as lactulose, and nonabsorbable antibiotics are utilized for treatment. Recently, lactulose and/or probiotic therapy have been shown to decrease serum ammonia levels [95]. Additionally, zinc supplementation is also effective in improving ammonia metabolism [96, 97]. 

### 7.4. Nutrition Therapy for LC with Obesity 

Obesity is a risk factor for various cancers and is closely associated with the incidence of HCC [69, 98]. In a large-scale Japanese study of LC patients, the percentage of PEM patients (BMI < 18.5 kg/m^2^) was 5.5%, while the percentage of obese patients (BMI > 25 kg/m^2^) was 28.3% [69]. A high BMI level as well as a high  *α*-fetoprotein level, a low albumin level, and complications of diabetes were associated with a significantly high hazard ratio for HCC in LC patients. Obese LC patients, even under diet therapy, were more likely to develop HCC than nonobese LC patients, but addition of oral BCAA granules to diet therapy reduced the incidence of HCC [69]. It has been shown that oral BCAA granules increase serum albumin levels independent of dietary intake [83]. The mechanism may be that BCAA improve insulin sensitivity in muscle, increase albumin in reduced form, and reduce oxidative stress [99–101]. Thus, in obese LC patients, oral BCAA treatment is recommended in addition to correcting nutritional intake. Excessive nutrients should be assessed in each patient, and the time course of nutritional parameters, such as serum albumin and lean body mass, should be determined for appropriate nutritional therapy. However, the level to which body weight should be reduced has not been examined sufficiently in obese LC patients. 

## 8. Conclusions

In NAFLD/NASH patients, elucidation of excessive nutrients by careful investigation of their dietary intake leads to better nutrition therapy. To obtain better results for antiviral therapy in CHC patients, nutritional care/support is a significant strategy. In CHC patients, low-iron diet therapy is effective for diminishing liver injury and preventing HCC. The nutritional state of LC patients has a wide spectrum. Therefore, nutrition therapy for LC patients should be planned after an assessment of their complications, nutritional state, and dietary intake. 

## References

[1] Assy N, Nasser G, Kamayse I (2008). Soft drink consumation linked with fatty liver in the absence of traditional risk factors. *Canadian Journal of Gastroenterology*.

[2] Ouyang X, Cirillo P, Sautin Y (2008). Fructose consumption as a risk factor for non-alcoholic fatty liver disease. *Journal of Hepatology*.

[3] Abid A, Taha O, Nseir W, Farah R, Grosovski M, Assy N (2009). Soft drink consumption is associated with fatty liver disease independent of metabolic syndrome. *Journal of Hepatology*.

[4] Toshimitsu K, Matsuura B, Ohkubo I (2007). Dietary habits and nutrient intake in non-alcoholic steatohepatitis. *Nutrition*.

[5] Yamazaki T, Nakamori A, Sasaki E, Wada S, Ezaki O (2007). Fish oil prevents sucrose-induced fatty liver but exacerbates high-safflower oil-induced fatty liver in ddY mice. *Hepatology*.

[6] Solga S, Alkhuraishe AR, Clark JM (2004). Dietary composition and nonalcoholic fatty liver disease. *Digestive Diseases and Sciences*.

[7] Musso G, Gambino R, De Michieli F (2003). Dietary habits and their relations to insulin resistance and postprandial lipemia in nonalcoholic steatohepatitis. *Hepatology*.

[8] Ha SK, Chae C (2010). Inducible nitric oxide distribution in the fatty liver of a mouse with high fat diet-induced obesity. *Experimental Animals*.

[9] Yasutake K, Nakamuta M, Shima Y (2009). Nutritional investigation of non-obese patients with non-alcoholic fatty liver disease: the significance of dietary cholesterol. *Scandinavian Journal of Gastroenterology*.

[10] Kainuma M, Fujimoto M, Sekiya N (2006). Cholesterol-fed rabbit as a unique model of nonalcoholic, nonobese, non-insulin-resistant fatty liver disease with characteristic fibrosis. *Journal of Gastroenterology*.

[11] Matsuzawa N, Takamura T, Kurita S (2007). Lipid-induced oxidative stress causes steatohepatitis in mice fed an atherogenic diet. *Hepatology*.

[12] Wouters K, van Gorp PJ, Bieghs V (2008). Dietary cholesterol, rather than liver steatosis, leads to hepatic inflammation in hyperlipidemic mouse models of nonalcoholic steatohepatitis. *Hepatology*.

[13] Higuchi N, Kato M, Shundo Y (2008). Liver X receptor in cooperation with SREBP-1c is a major lipid synthesis regulator in nonalcoholic fatty liver disease. *Hepatology Research*.

[14] Nakamuta M, Fujino T, Yada R (2009). Impact of cholesterol metabolism and the LXR*α*-SREBP-1c pathway on nonalcoholic fatty liver disease. *International Journal of Molecular Medicine*.

[15] Dixon JB, Bhathal PS, Hughes NR, O’Brien PE (2004). Nonalcoholic fatty liver disease: improvement in liver histological analysis with weight loss. *Hepatology*.

[16] Elias MC, Parise ER, Carvalho LD, Szejnfeld D, Netto JP (2010). Effect of 6-month nutritional intervention on non-alcoholic fatty liver disease. *Nutrition*.

[17] Capanni M, Calella F, Biagini MR (2006). Prolonged n-3 polyunsaturated fatty acid supplementation ameliorates hepatic steatosis in patients with non-alcoholic fatty liver disease: a pilot study. *Alimentary Pharmacology and Therapeutics*.

[18] Hasegawa T, Yoneda M, Nakamura K, Makino I, Terano A (2001). Plasma transforming growth factor-*β*1 level and efficacy of *α*-tocopherol in patients with non-alcoholic steatohepatitis: a pilot study. *Alimentary Pharmacology and Therapeutics*.

[19] Kojima SI, Watanabe N, Numata M, Ogawa T, Matsuzaki S (2003). Increase in the prevalence of fatty liver in Japan over the past 12 years: analysis of clinical background. *Journal of Gastroenterology*.

[20] Nonomura A, Enomoto Y, Takeda M (2005). Clinical and pathological features of non-alcoholic steatohepatitis. *Hepatology Research*.

[21] Enjoji M, Machida K, Kohjima M (2010). NPC1L1 inhibitor ezetimibe is a reliable therapeutic agent for non-obese patients with nonalcoholic fatty liver disease. *Lipids in Health and Disease*.

[22] Davies JP, Scott C, Oishi K, Liapis A, Ioannou YA (2005). Inactivation of NPC1L1 causes multiple lipid transport defects and protects against diet-induced hypercholesterolemia. *Journal of Biological Chemistry*.

[23] Deushi M, Nomura M, Kawakami A (2007). Ezetimibe improves liver steatosis and insulin resistance in obese rat model of metabolic syndrome. *FEBS Letters*.

[24] Ghafoorunissa, Ibrahim A, Rajkumar L, Acharya V (2005). Dietary (n-3) long chain polyunsaturated fatty acids prevent sucrose-induced insulin resistance in rats. *Journal of Nutrition*.

[25] Teran-Garcia M, Adamson AW, Yu G (2007). Polyunsaturated fatty acid suppression of fatty acid synthase (FASN): evidence for dietary modulation of NF-Y binding to the Fasn promoter by SREBP-1c. *Biochemical Journal*.

[26] Patek AJ, Post J (1941). Treatment of cirrhosis of the liver by a nutritious diet and supplements rich in vitamin B complex. *Journal of Clinical Investigation*.

[27] Iwasa M, Iwata K, Kaito M (2004). Efficacy of long-term dietary restriction of total calories, fat, iron, and protein in patients with chronic hepatitis C virus. *Nutrition*.

[28] Hamer C (2008). The impact of combination therapy with peginterferon alfa-2a and ribavirin on the energy intake and body weight of adult hepatitis C patients. *Journal of Human Nutrition and Dietetics*.

[29] Fioravante M, Alegre SM, Marin DM (2012). Weight loss and resting energy expenditure in patients with chronic hepatitis C before and during standard treatment. *Nutrition*.

[30] Fried MW (2002). Side effects of therapy of hepatitis C and their management. *Hepatology*.

[31] Manns MP, McHutchison JG, Gordon SC (2001). Peginterferon alfa-2b plus ribavirin compared with interferonalfa-2b plus ribavirin for initial treatment of chronic hepatitis C: a randomised trial. *Lancet*.

[32] Fried MW, Shiffman ML, Reddy KR (2002). Peginterferon alfa-2a plus ribavirin for chronic hepatitis C virus infection. *The New England Journal of Medicine*.

[33] Hayashi H, Takikawa T, Nishimura N, Yano M, Isomura T, Sakamoto N (1994). Improvement of serum aminotransferase levels after phlebotomy in patients with chronic active hepatitis C and excess hepatic iron. *American Journal of Gastroenterology*.

[34] Iwasa M, Kaito M, Ikoma J (2002). Dietary iron restriction improves aminotransferase levels in chronic hepatitis C patients. *Hepato-Gastroenterology*.

[35] Yano M, Hayashi H, Wakusawa S (2002). Long term effects of phlebotomy on biochemical and histological parameters of chronic hepatitis C. *American Journal of Gastroenterology*.

[36] Shintani Y, Fujie H, Miyoshi H (2004). Hepatitis C virus infection and diabetes: direct involvement of the virus in the development of insulin resistance. *Gastroenterology*.

[37] Bach N, Thung SN, Schaffner F (1992). The histological features of chronic hepatitis C and autoimmune chronic hepatitis: a comparative analysis. *Hepatology*.

[38] Lefkowitch JH, Schiff ER, Davis GL (1993). Pathological diagnosis of chronic hepatitis C: a multicenter comparative study with chronic hepatitis B. *Gastroenterology*.

[39] Romero-Gómez M, Del Mar Viloria M, Andrade RJ (2005). Insulin resistance impairs sustained response rate to peginterferon plus ribavirin in chronic hepatitis C patients. *Gastroenterology*.

[40] Poynard T, Ratziu V, McHutchison J (2003). Effect of treatment with peginterferon or interferon alfa-2b and ribavirin on steatosis in patients infected with hepatitis C. *Hepatology*.

[41] Hui JM, Sud A, Farrell GC (2003). Insulin resistance is associated with chronic hepatitis C and virus infection fibrosis progression. *Gastroenterology*.

[42] Hourigan LF, Macdonald GA, Purdie D (1999). Fibrosis in chronic hepatitis C correlates significantly with body mass index and steatosis. *Hepatology*.

[43] Eslam M, Aparcero R, Kawaguchi T (2011). Meta-analysis: Insulin resistance and sustained virological response in hepatitis C. *Alimentary Pharmacology and Therapeutics*.

[44] Deltenre P, Louvet A, Lemoine M (2011). Impact of insulin resistance on sustained response in HCV patients treated with pegylated interferon and ribavirin: a meta-analysis. *Journal of Hepatology*.

[45] Yano M, Ikeda M, Abe KI (2007). Comprehensive analysis of the effects of ordinary nutrients on hepatitis C virus RNA replication in cell culture. *Antimicrobial Agents and Chemotherapy*.

[46] Abu-Mouch S, Fireman Z, Jarchovsky J, Zeina AR, Assy N (2011). Vitamin D supplementation improves sustained virologic response in chronic hepatitis C, (genotype 1)-naive patients. *World Journal of Gastroenterology*.

[47] Yasutake K, Ichinose M, Bekki M (2012). Significance of dietary intake during combined peg-interferon plus ribavirin therapy for chronic hepatitis C: relationship between polyunsaturated fatty acid and early virologic response. *Journal of the Japan Dietetic Association*.

[48] Leu GZ, Lin TY, Hsu JTA (2004). Anti-HCV activities of selective polyunsaturated fatty acids. *Biochemical and Biophysical Research Communications*.

[49] Kapadia SB, Chisari FV (2005). Hepatitis C virus RNA replication is regulated by host geranylgeranylation and fatty acids. *Proceedings of the National Academy of Sciences of the United States of America*.

[50] Vitaglione P, Fogliano V, Stingo S, Scalfi L, Capraso N, Morisco F (2007). Development of a tomato-based food for special medical purposes as therapy adjuvant for patients with. *European Journal of Clinical Nutrition*.

[51] Seeff LB, Curto TM, Szabo G (2008). Herbal product use by persons enrolled in the hepatitis C antiviral long-term treatment against cirrhosis (HALT-C) trial. *Hepatology*.

[52] Bonkovsky HL, Banner BF, Rothman AL (1997). Iron and chronic viral hepatitis. *Hepatology*.

[53] Di Bisceglie AM, Axiotis CA, Hoofnagle JH, Bacon BR (1992). Measurements of iron status in patients with chronic hepatitis. *Gastroenterology*.

[54] Piperno A, D’Alba R, Fargion S (1995). Liver iron concentration in chronic viral hepatitis: a study of 98 patients. *European Journal of Gastroenterology and Hepatology*.

[55] Haque S, Chandra B, Gerber MA, Lok ASF (1996). Iron overload in patients with chronic hepatitis C: a clinicopathologic study. *Human Pathology*.

[56] Di Bisceglie AM, Bonkovsky HL, Chopra S (2000). Iron reduction as an adjuvant to interferon therapy in patients with chronic hepatitis C who have previously not responded to interferon: a multicenter, prospective, randomized, controlled trial. *Hepatology*.

[57] Erhardt A, Maschner-Olberg A, Mellenthin C (2003). HFE mutations and chronic hepatitis C: H63D and C282Y heterozygosity are independent risk factors for liver fibrosis and cirrhosis. *Journal of Hepatology*.

[58] Bonkovsky HL, Troy N, McNeal K (2002). Iron and HFE or TfR1 mutations as comorbid factors for development and progression of chronic hepatitis C. *Journal of Hepatology*.

[59] Fontana RJ, Israel J, LeClair P (2000). Iron reduction before and during interferon therapy of chronic hepatitis C: results of a multicenter, randomized, controlled trial. *Hepatology*.

[60] Kato J, Miyanishi K, Kobune M (2007). Long-term phlebotomy with low-iron diet therapy lowers risk of development of hepatocellular carcinoma from chronic hepatitis C. *Journal of Gastroenterology*.

[61] Mendenhall CL, Moritz TE, Roselle GA (1993). A study of oral nutritional support with oxandrolone in malnourished patients with alcoholic hepatitis: results of a Department of Veterans Affairs cooperative study. *Hepatology*.

[62] Richardson RA, Davidson HI, Hinds A, Cowan S, Rae P, Garden OJ (1999). Influence of the metabolic sequelae of liver cirrhosis on nutritional intake. *American Journal of Clinical Nutrition*.

[63] Campillo B, Richardet JP, Scherman E, Bories PN (2003). Evaluation of nutritional practice in hospitalized cirrhotic patients: results of a prospective study. *Nutrition*.

[64] Merli M (1994). Nutritional status in cirrhosis. *Journal of Hepatology*.

[65] Tajika M, Kato M, Mohri H (2002). Prognostic value of energy metabolism in patients with viral liver cirrhosis. *Nutrition*.

[66] Owen OE, Reichle FA, Mozzoli MA (1981). Hepatic, gut, and renal substrate flux rates in patients with hepatic cirrhosis. *Journal of Clinical Investigation*.

[67] Nielsen K, Kondrup J, Martinsen L, Stilling B, Wikman B (1993). Nutritional assessment and adequacy of dietary intake in hospitalized patients with alcoholic liver cirrhosis. *British Journal of Nutrition*.

[68] Campillo B, Bories PN, Leluan M, Pornin B, Devanlay M, Fouet P (1995). Short-term changes in energy metabolism after 1 month of a regular oral diet in severely malnourished cirrhotic patients. *Metabolism: Clinical and Experimental*.

[69] Muto Y, Sato S, Watanabe A (2006). Overweight and obesity increase the risk for liver cancer in patients with liver cirrhosis and long-term oral supplementation with branched-chain amino acid granules inhibits liver carcinogenesis in heavier patients with liver cirrhosis. *Hepatology Research*.

[70] Yasutake K, Bekki M, Ichinose M (2012). Assessing current nutritional status of patients with HCV-related liver cirrhosis in the compensated stage. *Asia Pacific Journal of Clinical Nutrition*.

[71] Plauth M, Cabré E, Riggio O (2006). ESPEN guidelines on enteral nutrition: liver disease. *Clinical Nutrition*.

[72] Kato M, Miwa Y, Tajika M, Hiraoka T, Muto Y, Moriwaki H (1998). Preferential use of branched-chain amino acids as an energy substrate in patients with liver cirrhosis. *Internal Medicine*.

[73] Shiraki M, Shimomura Y, Miwa Y (2005). Activation of hepatic branched-chain *α*-keto acid dehydrogenase complex by tumor necrosis factor-*α* in rats. *Biochemical and Biophysical Research Communications*.

[74] Swart GR, Zillikens MC, Van Vuure JK, Van den Berg JWO (1989). Effect of a late evening meal on nitrogen balance in patients with cirrhosis of the liver. *British Medical Journal*.

[75] Zillikens MC, Van Den Berg JWO, Wattimena JLD, Rietveld T, Swart GR (1993). Nocturnal oral glucose supplementation. The effects on protein metabolism in cirrhotic patients and in healthy controls. *Journal of Hepatology*.

[76] Yamauchi M, Takeda K, Sakamoto K, Ohata M, Toda G (2001). Effect of oral branched chain amino acid supplementation in the late evening on the nutritional state of patients with liver cirrhosis. *Hepatology Research*.

[77] Tsuchiya M, Sakaida I, Okamoto M, Okita K (2005). The effect of a late evening snack in patients with liver cirrhosis. *Hepatology Research*.

[78] Nakaya Y, Okita K, Suzuki K (2007). BCAA-enriched snack improves nutritional state of cirrhosis. *Nutrition*.

[79] Aoyama K, Tsuchiya M, Mori K (2007). Effect of a late evening snack on outpatients with liver cirrhosis. *Hepatology Research*.

[80] Chang WK, Chao YC, Tang HS, Lang HF, Hsu CT (1997). Effects of extra-carbohydrate supplementation in the late evening on energy expenditure and substrate oxidation in patients with liver cirrhosis. *Journal of Parenteral and Enteral Nutrition*.

[81] Yamanaka-Okumura H, Nakamura T, Takeuchi H (2006). Effect of late evening snack with rice ball on energy metabolism in liver cirrhosis. *European Journal of Clinical Nutrition*.

[82] Miwa Y, Shiraki M, Kato M (2000). Improvement of fuel metabolism by nocturnal energy supplementation in patients with liver cirrhosis. *Hepatology Research*.

[83] Yatsuhashi H, Ohnishi Y, Nakayama S (2011). Anti-hypoalbuminemic effect of branched-chain amino acid granules in patients with liver cirrhosis is independent of dietary energy and protein intake. *Hepatology Research*.

[84] Marchesini G, Bianchi G, Merli M (2003). Nutritional supplementation with branched-chain amino acids in advanced cirrhosis: a double-blind, randomized trial. *Gastroenterology*.

[85] Poon RTP, Yu WC, Fan ST, Wong J (2004). Long-term oral branched chain amino acids in patients undergoing chemoembolization for hepatocellular carcinoma: a randomized trial. *Alimentary Pharmacology and Therapeutics*.

[86] Kato A, Suzuki K (2004). How to select BCAA preparations. *Hepatology Research*.

[87] Imano E, Kanda T, Nakatani Y (1999). Impaired splanchnic and peripheral glucose uptake in liver cirrhosis. *Journal of Hepatology*.

[88] Petrides AS, Stanley T, Matthews DE, Vogt C, Bush AJ, Lambeth H (1998). Insulin resistance in cirrhosis: prolonged reduction of hyperinsulinemia normalizes insulin sensitivity. *Hepatology*.

[89] Barkoukis H, Fiedler KM, Lerner E (2002). A combined high-fiber, low-glycemic index diet normalizes glucose tolerance and reduces hyperglycemia and hyperinsulinemia in adults with hepatic cirrhosis. *Journal of the American Dietetic Association*.

[90] Jenkins DJA, Shapira N, Greenberg G (1989). Low glycemic index foods and reduced glucose, amino acid, and endocrine responses in cirrhosis. *American Journal of Gastroenterology*.

[91] Gentile S, Turco S, Guarino G (2001). Effect of treatment with acarbose and insulin in patients with non-insulin-dependent diabetes mellitus associated with non-alcoholic liver cirrhosis. *Diabetes, Obesity and Metabolism*.

[92] Zillikens MC, Swart GR, Van den Berg JWO, Wilson JHP (1989). Effects of the glucosidase inhibitor acarbose in patients with liver cirrhosis. *Alimentary Pharmacology and Therapeutics*.

[93] Marchesini G, Bugianesi E, Ronchi M, Flamia R, Thomaseth K, Pacini G (1998). Zinc supplementation improves glucose disposal in patients with cirrhosis. *Metabolism: Clinical and Experimental*.

[94] Córdoba J, López-Hellín J, Planas M (2004). Normal protein diet for episodic hepatic encephalopathy: results of a randomized study. *Journal of Hepatology*.

[95] Sharma P, Sharma BC, Puri V, Sarin SK (2008). An open-label randomized controlled trial of lactulose and probiotics in the treatment of minimal hepatic encephalopathy. *European Journal of Gastroenterology and Hepatology*.

[96] Reding P, Duchateau J, Bataille C (1984). Oral zinc supplementation improves hepatic encephalopathy. Results of a randomised controlled trial. *Lancet*.

[97] Takuma Y, Nousot K, Makino Y, Hayashi M, Takahashi H (2010). Clinical trial: oral zinc in hepatic encephalopathy. *Alimentary Pharmacology and Therapeutics*.

[98] Calle EE, Rodriguez C, Walker-Thurmond K, Thun MJ (2003). Overweight, obesity, and mortality from cancer in a prospectively studied cohort of U.S. Adults. *The New England Journal of Medicine*.

[99] Nishitani S, Takehana K, Fujitani S, Sonaka I (2005). Branched-chain amino acids improve glucose metabolism in rats with liver cirrhosis. *American Journal of Physiology*.

[100] Fukushima H, Miwa Y, Shiraki M (2007). Oral branched-chain amino acid supplementation improves the oxidized/ reduced albumin ratio in patients with liver cirrhosis. *Hepatology Research*.

[101] Ohno T, Tanaka Y, Sugauchi F (2008). Suppressive effect of oral administration of branched-chain amino acid granules on oxidative stress and inflammation in HCV-positive patients with liver cirrhosis. *Hepatology Research*.

